# Supplementary material for: Crystal structure of the α1B-adrenergic receptor reveals molecular determinants of selective ligand recognition
Source: Nat Commun. 2022 Jan 19;13:382. doi: 10.1038/s41467-021-27911-3 (PMC8770593; doi:10.1038/s41467-021-27911-3)
Supplement: Supplementary file 1 — Supplementary Information [file 41467_2021_27911_MOESM1_ESM.pdf]

Supplementary Information

Crystal structure of the α_{1B}-adrenergic receptor reveals molecular determinants of selective ligand recognition

Mattia Deluigi, Lena Morstein, Matthias Schuster, Christoph Klenk, Lisa Merklinger, Riley R. Cridge, Lazarus A. de Zhang, Alexander Klipp, Santiago Vacca, Tasneem M. Vaid, Peer R. E. Mittl, Pascal Egloff, Stefanie A. Eberle, Oliver Zerbe, David K. Chalmers, Daniel J. Scott*, Andreas Plückthun*

These authors contributed equally: Lena Morstein, Matthias Schuster.

*Corresponding authors. Email: daniel.scott@florey.edu.au; plueckthun@bioc.uzh.ch

Supplementary Figures

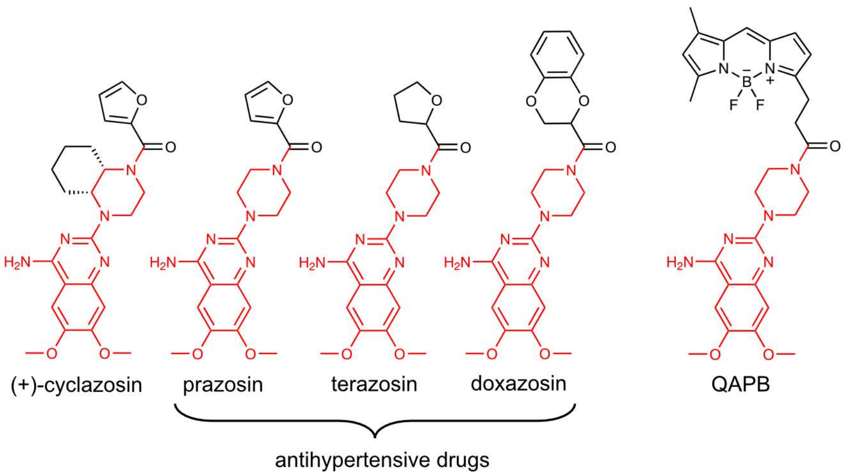

Supplementary Fig. 1. Chemical structure of (+)-cyclazosin and of analogs clinically used as antihypertensive agents. The shared piperazinyl 4-amino-6,7-dimethoxyquinazoline scaffold is highlighted in red. The unprotonated forms are depicted here, as these are commonly drawn, while the protonated forms of cyclazosin, prazosin, and QAPB (quinazolinyl piperazine BODIPY) are shown in the main text (see Fig. 1a and Fig. 5a–c), as these are the predominant forms under crystallization conditions (pH 6) and at physiological pH (see main text). QAPB is used as the fluorescent tracer ligand in competition ligand-binding experiments (see below).

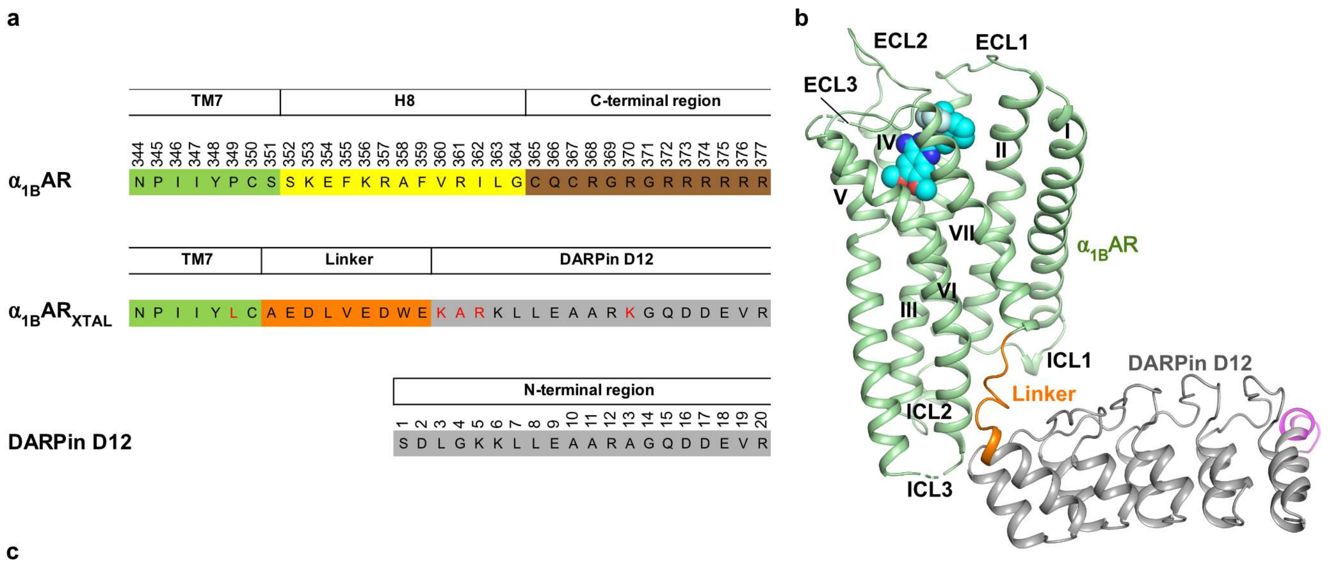

c

GPGSSTLPQLDITRAISVGLVLGAFILFAIVGNILVILSVACNRHLRTPTNYFIVNLAMADLLLCFTVLPFSAALEVLGYWVLGRT
FCDIWAAMDVLCCTASILSLCAISIDRYIGVRYYLQYPTLVTRRKAILALLCVWVLSTVISIGPLLVWKEPAPNYDKVCGVTEEPF
YALFSSLGSFYIPLAVILVMYCRVYIVAKRTTKNLEAGVMKEMSNSFKFSREKKAAKMLGIVVGMFILCWLPFFIALPLGSLFSTL
KPPDAVFKVLLWLGYFNSCLNPIIYLCAEDLVEDWEKARKLLEAARKGQDDEVRILLANGADVNTADETGFTPLHLAAWEGHLGIV
EVLLKNGADVNANDERGHTPLHLAAYTGHLEIVEVLLKNGAGVNATDVIGTAPLHLAAMWGHLEIVEVLLKNGADVNAQDKFGKTP
FDLAIDNGNEDIAEVLQKAATRELEVLFQ

Supplementary Fig. 2. Fusion of DARPin D12 to the C-terminal end of TM7 of α1BAR. a Fusion site. Top row: C-terminal end of TM7 of α1BAR (pale green), helix 8 (H8, yellow), and beginning of C-terminal region (brown) in wt α1BAR. Bottom row: N-terminal region of DARPin D12 (gray). Middle row: crystallized fusion construct. It is a fusion of the C-terminal end of TM7 of α1BAR (pale green) via a linker (orange) to DARPin D12 (gray). This construct harbors the P349→L mutation (see Methods), highlighted in red. In addition, the first two N-terminal residues of DARPin D12 were deleted, and four mutations were introduced, also highlighted in red. **b** Overview structure of α1BARXTAL bound to (+)-cyclazosin. The protein is shown as cartoon; (+)-cyclazosin is depicted as van der Waals spheres. Colors are as in panel a; (+)-cyclazosin is colored in cyan, with the two orientations observed for its furan-2-yl-methanone substituent colored in cyan and pale cyan, respectively. Of note, the C-terminal region of the crystallized fusion construct (see Methods), colored in magenta, turned out to be partially α-helical and established crystal contacts (cf. Supplementary Fig. 4d). ECL, extracellular loop; ICL, intracellular loop. **c** Sequence of α1BARXTAL (see Methods for details). Colors are as in panels a and b, i.e., α1BAR is colored in pale green, DARPin D12 in gray, the linker between α1BAR and DARPin D12 in orange, and the C-terminal region of α1BARXTAL in magenta. All twelve mutations within α1BAR and all six mutations within DARPin D12 are highlighted in red.

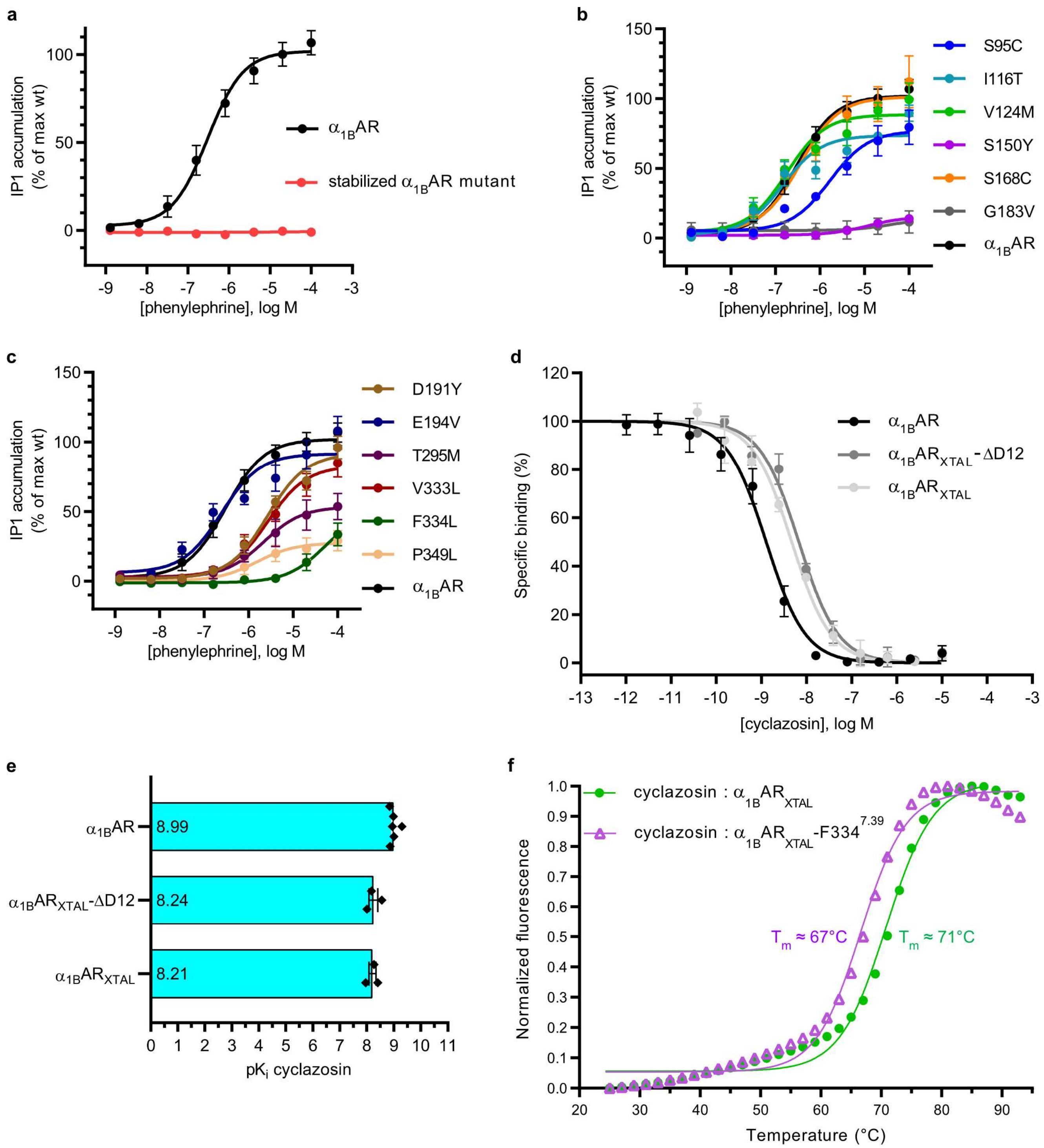

Supplementary Fig. 3. Pharmacological and biophysical characterization of stabilized α_{1B}AR constructs. a Agonist-induced G_q signaling in cells expressing wild-type α_{1B}AR and the stabilized α_{1B}AR mutant used in this study (the mutations are listed in Supplementary Table 1). Data are shown as mean values ± standard error of the mean (SEM); the number of independent experiments is reported in Supplementary Table 2. **b**, **c** Impact of each of the 12 mutations in $\alpha_{1B}AR_{XTAL}$ on agonist-induced G_q signaling. The constructs harbor the individual mutation indicated. Data are shown as mean values ± SEM; the number of independent experiments and the 95% confidence interval of the mean are reported in Supplementary Table 2. **d** Competition ligand-binding curves with QAPB as the tracer and cyclazosin as the competitor for wild-type α_{1B}AR, the stabilized receptor variant without DARPin D12 ($\alpha_{1B}AR_{XTAL}$-ΔD12) or with DARPin D12 fusion ($\alpha_{1B}AR_{XTAL}$). Data are shown as mean values ± standard deviation (SD) from either 6 independent experiments performed in technical triplicates (wild-type α_{1B}AR) or 3 independent experiments performed in technical duplicates ($\alpha_{1B}AR_{XTAL}$-ΔD12 and $\alpha_{1B}AR_{XTAL}$). **e** pK_i values of cyclazosin corresponding to panel d. Data are shown as mean values

± SEM. The underlying data points are depicted as black diamonds. The 95% confidence interval of the mean is as follows in square parentheses: α_{1B}AR [8.82 to 9.16]; $\alpha_{1B}AR_{XTAL}$-ΔD12 [7.55 to 8.94]; $\alpha_{1B}AR_{XTAL}$ [7.65 to 8.77]. Differences in affinities were evaluated by a statistical test as detailed in Supplementary Table 7. **f** CPM-based thermostability assay[1] of cyclazosin-bound $\alpha_{1B}AR_{XTAL}$ and $\alpha_{1B}AR_{XTAL}$-F334$^{7.39}$, the latter bearing the L334$^{7.39}$→F back-mutation (Ballesteros-Weinstein numbering denoted in superscript[2]). Data from a representative experiment are shown. The apparent melting temperature (T_m) was estimated from these data using non-linear regression with *GraphPad Prism* 8.4.3. Source data are provided as a Source Data file.

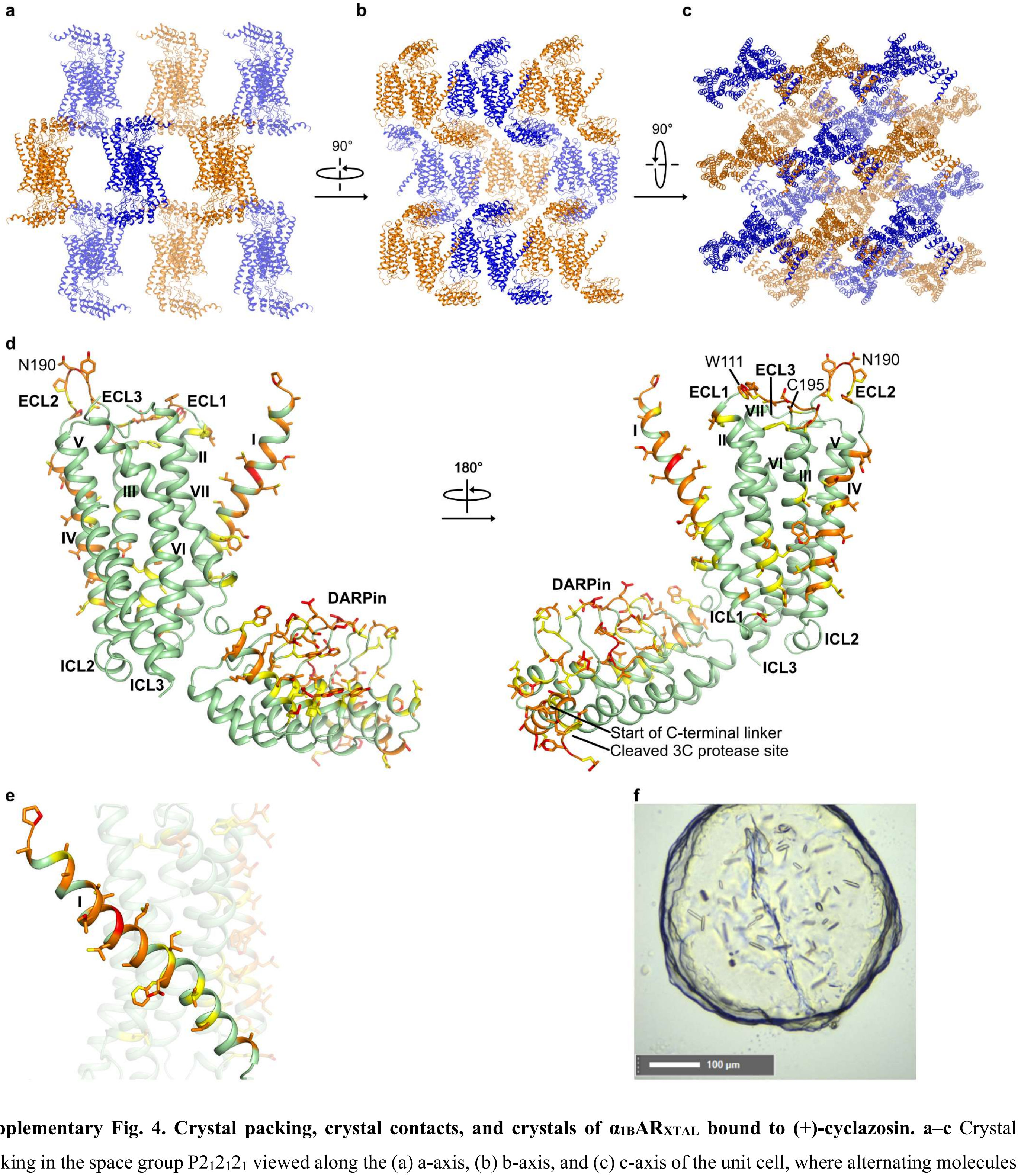

Supplementary Fig. 4. Crystal packing, crystal contacts, and crystals of $\alpha_{1B}AR_{XTAL}$ bound to (+)-cyclazosin. a–c Crystal packing in the space group $P2_12_12_1$ viewed along the (a) a-axis, (b) b-axis, and (c) c-axis of the unit cell, where alternating molecules are colored in blue and orange. Molecules lying in the backplane are displayed in lighter shades. The ligand has been omitted for clarity. **d** Residues involved in crystal contacts. The protein backbone is depicted as cartoon; residues are shown as sticks. Residues involved in contacts formed within ≤ 3.5 Å are highlighted in red, > 3.5 Å but ≤ 5 Å in orange, while atoms outside of the 5 Å cut-off but belonging to a residue involved in crystal contacts are highlighted in yellow. Regions not involved in crystal contacts are colored in pale green. **e** Close-up view of the crystallization interface formed by TM1. Representation and colors are as in panel d. **f** Crystals of $\alpha_{1B}AR_{XTAL}$ grown in LCP. Photo Credit: Mattia Deluigi using a Rock Imager 1000 (Formulatrix), Department of Biochemistry, University of Zurich.

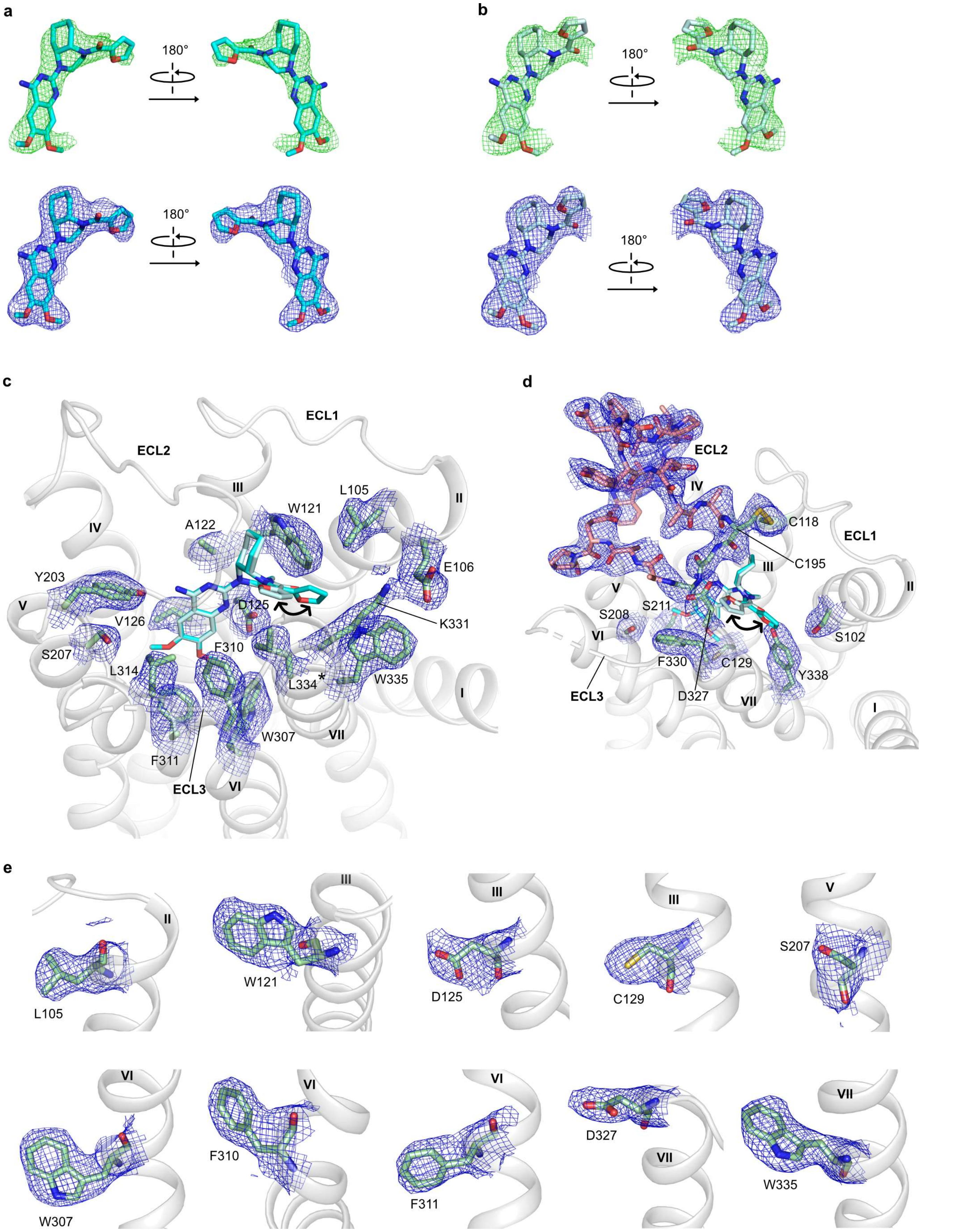

Supplementary Fig. 5. Electron density of (+)-cyclazosin and of $\alpha_{1B}AR_{XTAL}$ in key receptor regions. a, **b** Electron density maps of (+)-cyclazosin. The two orientations modeled for the furan-2-yl-methanone substituent are shown in panels a and b, respectively. Fo–Fc maps are depicted as green mesh contoured at 2.5 σ (panel a) or 2.0 σ (panel b). The Fo–Fc maps were computed before the ligand was modeled in the electron density (i.e., only the protein and solvent had been modeled). 2Fo–Fc maps are depicted as blue

mesh contoured at 1.0 σ; (+)-cyclazosin is depicted as sticks either in cyan (panel a) or pale cyan (panel b). Oxygen and nitrogen atoms are shown in red and blue, respectively. **c**, **d** 2Fo–Fc electron density maps of residues belonging to the ligand-binding pocket and ECL2. **e** More detailed view of the 2Fo–Fc electron density maps of selected residues belonging to the ligand-binding pocket (cf. panels c and d). The receptor backbone is shown as cartoon in light gray; receptor residues delineating the ligand-binding pocket are shown as sticks in pale green; in panel d, ECL2 is shown as sticks in salmon pink. (+)-Cyclazosin is depicted as in panels a and b, with the two orientations of its furan-2-yl-methanone substituent indicated by a black curved arrow. The asterisk indicates the F334→L mutation present in $\alpha_{1B}AR_{XTAL}$.

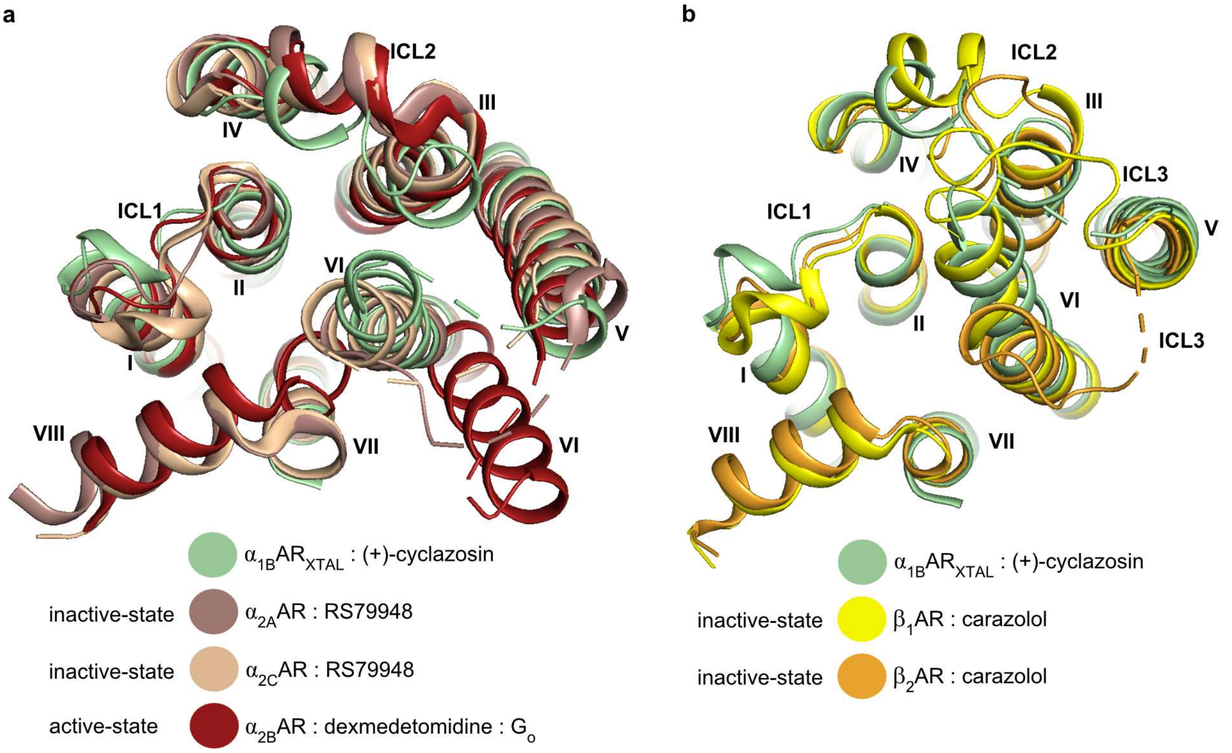

Supplementary Fig. 6. Arrangement of the 7TM bundle at the intracellular side in $\alpha_{1B}AR_{XTAL}$ compared to other ARs. a Superposition of $\alpha_{1B}AR_{XTAL}$ bound to (+)-cyclazosin with RS79948-bound $\alpha_{2A}AR$ (PDB ID: 6KUX[3]) and $\alpha_{2C}AR$ (PDB ID: 6KUW[4]), and with the active-state agonist-bound $\alpha_{2B}AR$-G_o complex (PDB ID: 6K41[5]), viewed from the intracellular side. The receptor backbone is depicted as cartoon. Ligands, fusion proteins, and G protein have been omitted for clarity. **b** Superposition of $\alpha_{1B}AR_{XTAL}$ bound to (+)-cyclazosin with carazolol-bound β_1AR (PDB ID: 2YCW[6]) and β_2AR (PDB ID: 2RH1[7]), viewed from the intracellular side.

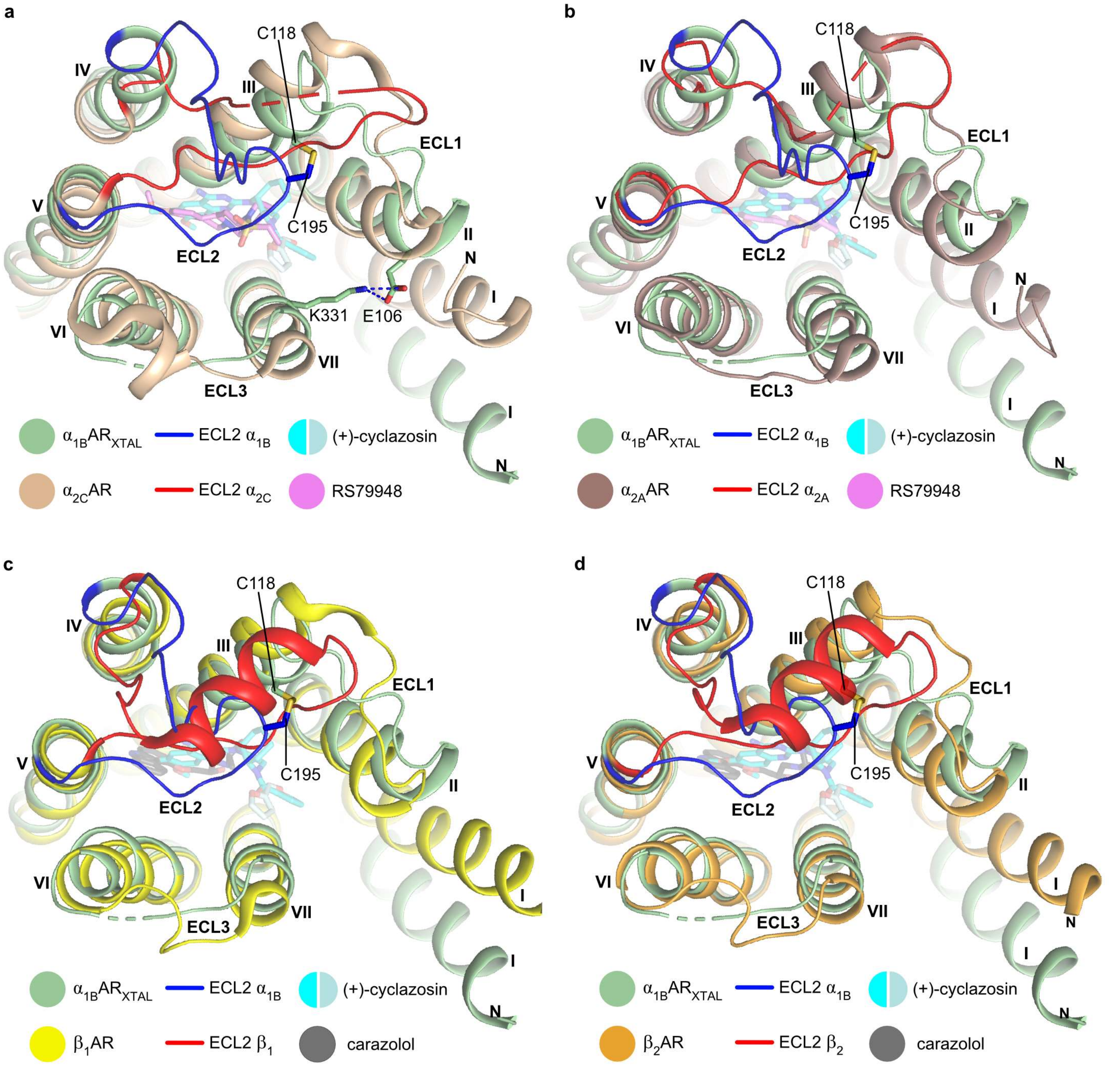

Supplementary Fig. 7. Arrangement of the 7TM bundle at the extracellular side in $\alpha_{1B}AR_{XTAL}$ compared to other ARs. a–d Superposition of $\alpha_{1B}AR_{XTAL}$ bound to (+)-cyclazosin with (a) RS79948-bound α_{2C}AR (PDB ID: 6KUW[4]), (b) RS79948-bound α_{2A}AR (PDB ID: 6KUX[3]), (c) carazolol-bound β_1AR (PDB ID: 2YCW[6]), and (d) carazolol-bound β_2AR (PDB ID: 2RH1[7]), all viewed from the extracellular side. The receptor backbone is depicted as cartoon; receptor residues are shown as sticks; ligands are shown as transparent sticks. Oxygen, nitrogen, and sulfur atoms are depicted in red, blue, and yellow, respectively.

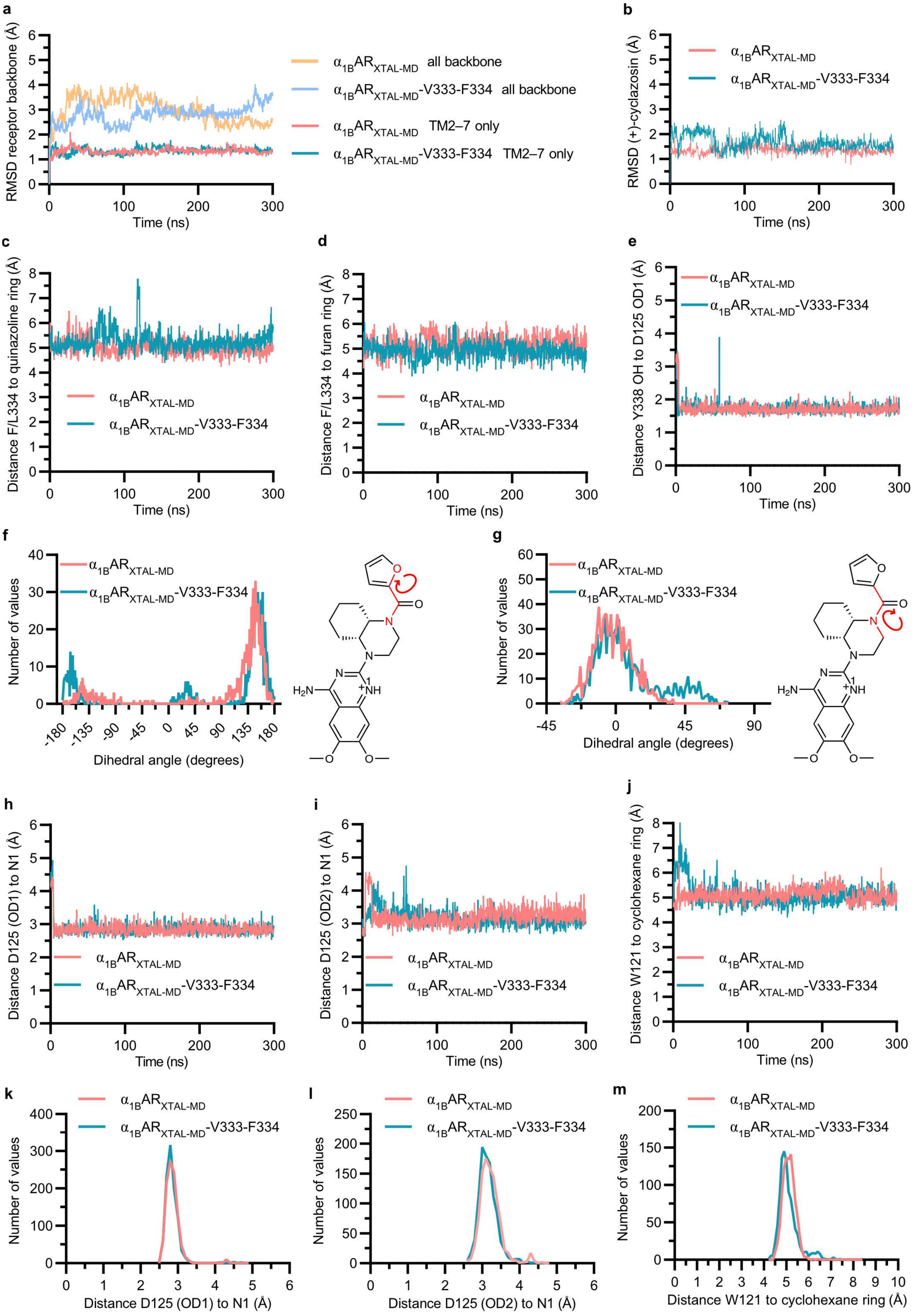

Supplementary Fig. 8. MD simulations. The DARPin D12 fusion was excised in the simulations. **a**, **b** Structural stability of (a) the receptor and (b) (+)-cyclazosin throughout the simulations. The trajectories were aligned using all backbone atoms of TM2–7. For

the receptor, root-mean-square deviation (RMSD) values were then calculated on either all backbone atoms or on backbone atoms of TM2–7 only. The loops and TM1 are conformationally more flexible than TM2–7. For (+)-cyclazosin, RMSD values were calculated on all atoms. **c**, **d** Distance between F/L334$^{7.39}$ and either (c) the quinazoline ring or (d) the furan ring of (+)-cyclazosin throughout the simulations. Distances were measured between ring centers, or between the ring centers and the center of mass of Cδ1 and Cδ2 of L334$^{7.39}$. **e** Distance between OD1 of D125$^{3.32}$ and OH of Y338$^{7.43}$ throughout the simulations. **f**, **g** Frequency distribution of dihedrals in (+)-cyclazosin, showing a very similar distribution in the presence and absence of the back-mutations. The plotted dihedrals refer to the set of four consecutively bonded atoms highlighted in red in the chemical structure on the right of the corresponding plot. **h**–**j** Additional receptor-ligand distances throughout the simulations. **k**–**m** Frequency distribution of receptor-ligand distances corresponding to panels h–j. Source data are provided as a Source Data file.

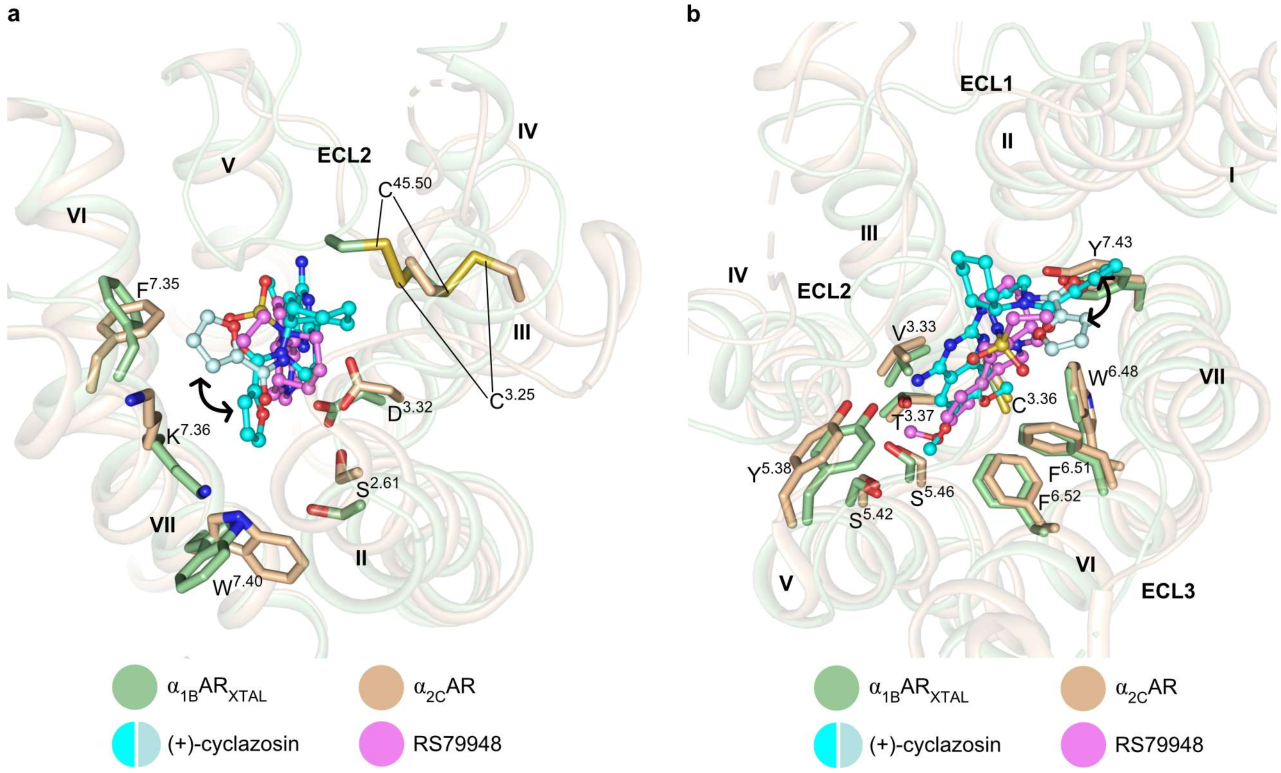

Supplementary Fig. 9. Side-chain conformations within the ligand-binding pockets of $\alpha_{1B}AR_{XTAL}$ and $\alpha_{2C}AR$-RS79948. Superposition of $\alpha_{1B}AR_{XTAL}$ bound to (+)-cyclazosin with $\alpha_{2C}AR$-RS79948 (PDB ID: 6KUW[4]). **a** Conserved residues displaying conformational or positional side-chain deviations ($S^{2.61}$, $D^{3.32}$, $C^{3.25}$, $C^{45.50}$, $F^{7.35}$, $K^{7.36}$, and $W^{7.40}$). The N-terminus of $\alpha_{2C}AR$ was omitted for clarity. Residues are depicted as sticks; ligands are shown in ball-and-stick representation. A black curved arrow indicates the two orientations observed for the furan-2-yl-methanone moiety of (+)-cyclazosin. Oxygen, nitrogen, and sulfur atoms are depicted in red, blue, and yellow, respectively. **b** Conserved residues displaying similar conformations ($V^{3.33}$, $C^{3.36}$, $T^{3.37}$, $Y^{5.38}$, $S^{5.42}$, $S^{5.46}$, $W^{6.48}$, $F^{6.51}$, $F^{6.52}$, and $Y^{7.43}$). Representation and colors are as in panel a.

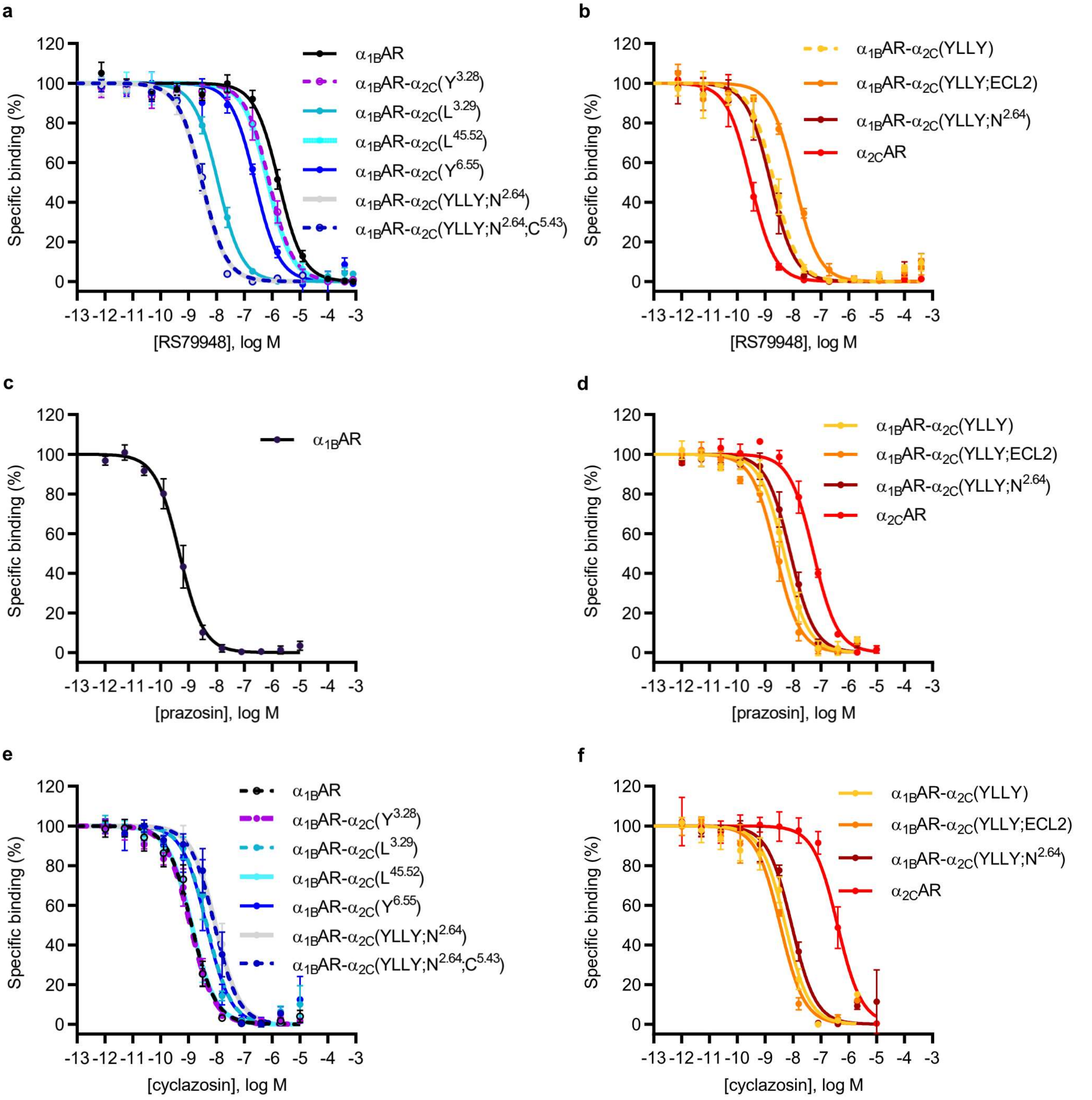

Supplementary Fig. 10. Competition ligand-binding for α_{1B}AR, α_{2C}AR, and chimeric α_{1B}AR-α_{2C} mutants. a, **b** RS79948. **c**, **d** Prazosin. **e**, **f** Cyclazosin. 2 nM QAPB was used as the tracer ligand in panels a, c, and e, whereas 50 nM QAPB was used in panels b, d, and f. Data are shown as mean values ± SD from 3–8 independent experiments performed in technical triplicates. The exact n values are reported in Supplementary Table 6. Source data are provided as a Source Data file.

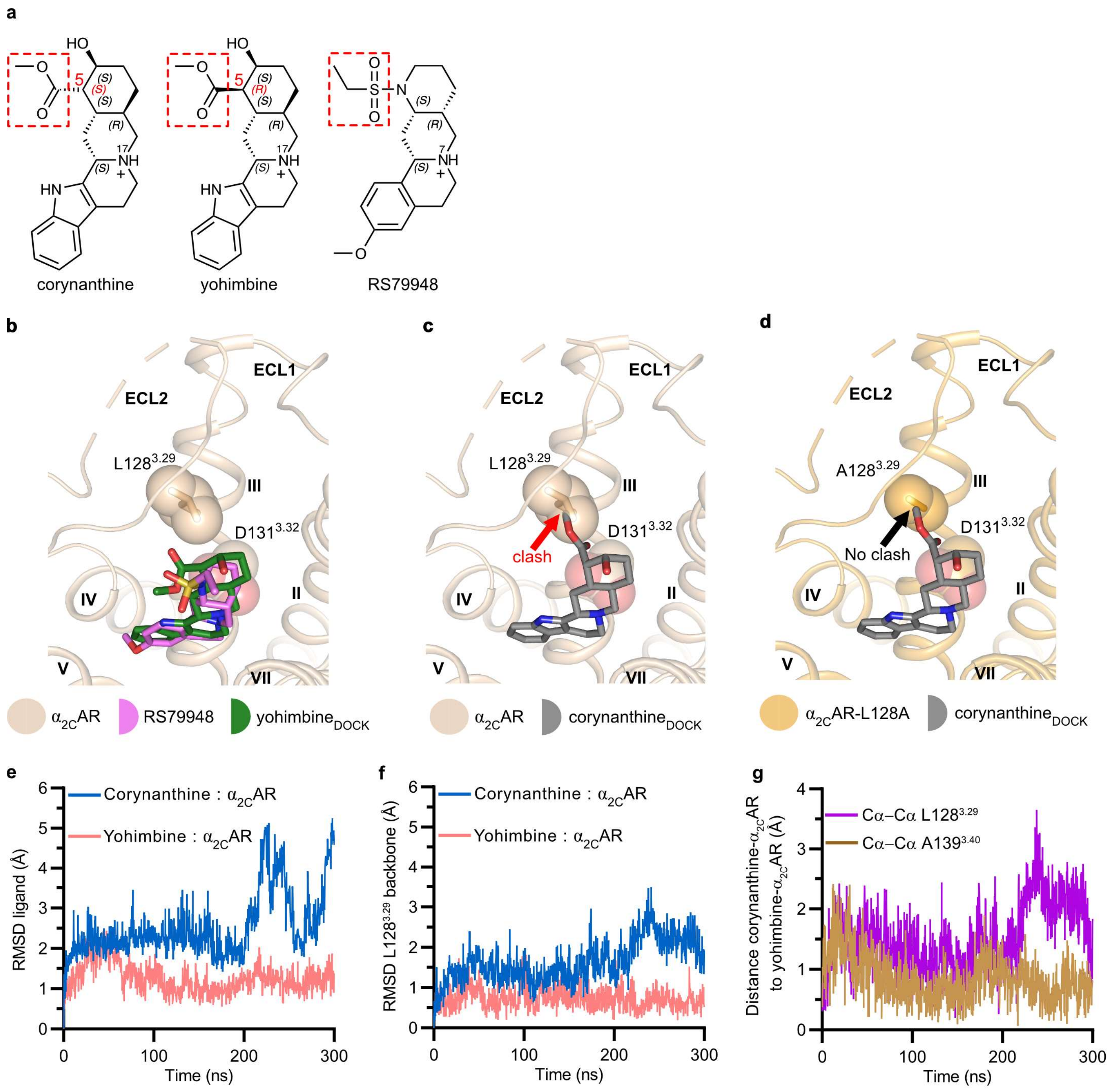

Supplementary Fig. 11. Docking suggests that residue 3.29 underlies stereoselective ligand recognition in αARs. a Chemical structure of the indicated ligands. The relevant stereocenter, C5, and its absolute configuration are highlighted in red. A dashed red box highlights the bulky CO_2CH_3 group linked to this stereocenter, as well as the $SO_2CH_2CH_3$ moiety of RS79948. **b** Docking of yohimbine to α_{2C}AR (PDB ID: 6KUW[4]). The bulky CO_2CH_3 group of yohimbine and the $SO_2CH_2CH_3$ group of RS79948 point in the same direction, i.e., away from L128$^{3.29}$. **c**, **d** Initial docking experiments using a rigid α_{2C}AR (PDB ID: 6KUW[4]) suggested that if corynanthine were to adopt a very similar conformation and binding pose as observed for yohimbine and RS79948, its CO_2CH_3 group would likely clash with L128$^{3.29}$ (panel c), but not with A128$^{3.29}$ (panel d). As docking of corynanthine to a rigid α_{2C}AR (bearing L128$^{3.29}$) failed to yield energetically favorable poses where N17 interacts with the side chain of D131$^{3.32}$, we docked this ligand to α_{2C}AR-L128$^{3.29}$→A (based on PDB ID: 6KUW[4]). Docking resulted in a binding mode where N17 and D131$^{3.32}$ are in interaction distance, and the polycyclic ring system is positioned similarly as observed for yohimbine and RS79948. A black arrow indicates that no clash is seen between the CO_2CH_3 group of corynanthine and A128$^{3.29}$. Corynanthine$_{DOCK}$ was then superimposed with α_{2C}AR (PDB ID: 6KUW[4]) to reveal a steric clash between its CO_2CH_3 group and L128$^{3.29}$, indicated by a red arrow. Ligands

are depicted as sticks; receptor residues are depicted as sticks and as van der Waals spheres. Oxygen, nitrogen, and sulfur atoms are depicted in red, blue, and yellow, respectively. **e–g** To assess binding of yohimbine and corynanthine in a less rigid system, we carried out MD simulations. The binding of yohimbine in α_{2C}AR remained very stable. In contrast, corynanthine sampled different conformations and positions within the pocket to avoid a steric clash with L128$^{3.29}$; however, none resulted in stable binding (panel e, and Supplementary Movie 1). Furthermore, the backbone of L128$^{3.29}$ was pushed back during the simulation with corynanthine but not in the simulation with yohimbine (panels f and g, and Supplementary Movie 1). This suggests again that the bulky side chain of L128$^{3.29}$ is involved in the unstable binding of corynanthine in α_{2C}AR. Source data are provided as a Source Data file.

a

	Ballesteros-Weinstein															
	23.50	3.28	3.32	3.33	3.36	3.37	3.40	5.46	5.47	6.44	6.48	6.51	6.52	7.35	7.39	7.43
DRD2	W100	F110	D114	V115	C118	T119	I122	S197	F198	F382	W386	F389	F390	Y408	T412	Y416
α_{1A}AR	W	W	D	V	C	T	I	S	F	F	W	F	F	F	F	Y
α_{1B}AR	W111	W121	D125	V126	C129	T130	I133	S211	F212	F303	W307	F310	F311	F330	F334	Y338
α_{1D}AR	W	W	D	V	C	T	I	S	F	F	W	F	F	F	F	Y

b

	Ballesteros-Weinstein															
	2.64	23.50	3.28	3.29	3.32	3.36	3.40	45.52	5.46	5.47	6.44	6.48	6.51	6.52	7.35	7.43
DRD2	L94	W100	F110	V111	D114	C118	I122	I184	S197	F198	F382	W386	F389	F390	Y408	Y416
α_{1A}AR	F	W	W	A	D	C	I	I	S	F	F	W	F	F	F	Y
α_{1B}AR	L105	W111	W121	A122	D125	C129	I133	V197	S211	F212	F303	W307	F310	F311	F330	Y338
α_{1D}AR	M	W	W	A	D	C	I	I	S	F	F	W	F	F	F	Y

Supplementary Fig. 12. Conservation of residues involved in risperidone and haloperidol binding to the D2 dopamine receptor (DRD2) across α$_1$ARs. a, **b** Residues involved in (a) risperidone and (b) haloperidol binding to DRD2 and their conservation in α$_1$ARs. Residues interacting with risperidone and haloperidol are as defined in supplementary references 8 and 9, respectively. Aromatic residues are highlighted in orange, hydrophobic residues in yellow, polar residues in green, Cys in yellow-green, acidic residues in red.

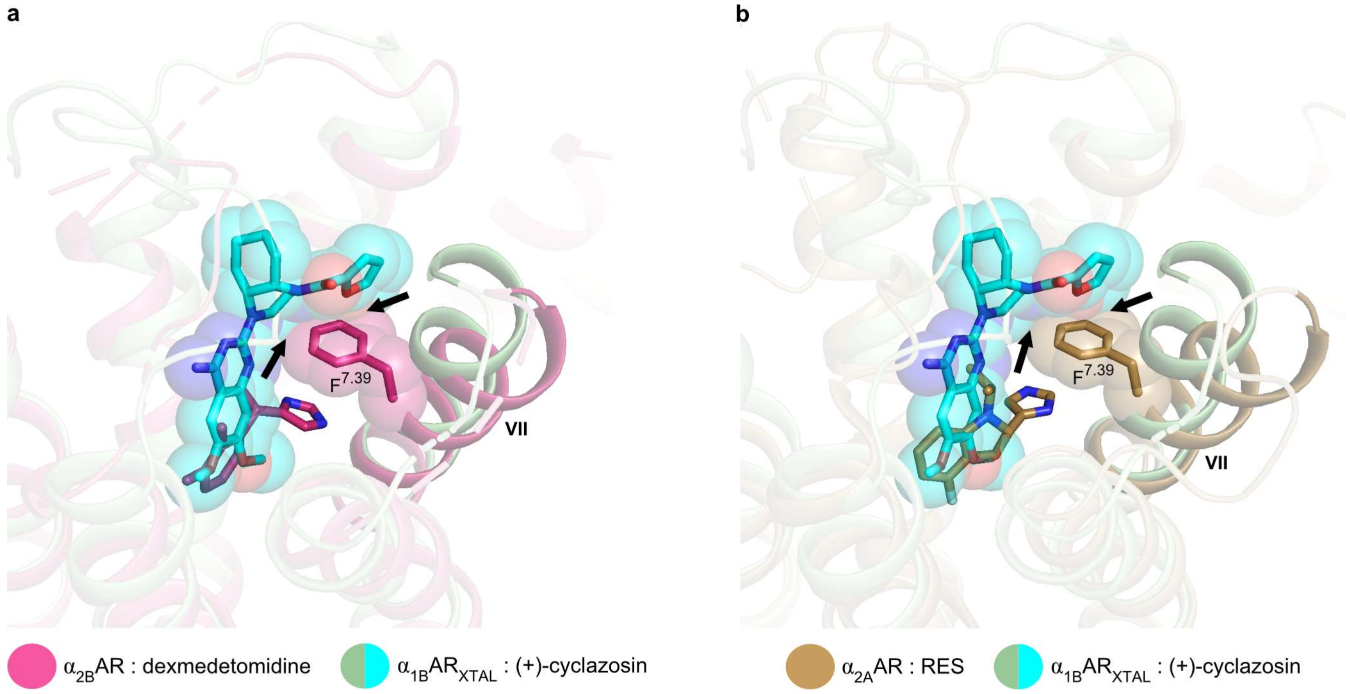

Supplementary Fig. 13. Inverse agonist (+)-cyclazosin possibly prevents $F^{7.39}$ from adopting a rotamer critical for receptor activation. a, **b** Superposition of $\alpha_{1B}AR_{XTAL}$ bound to (+)-cyclazosin with (a) agonist-bound active-state α_{2B}AR (PDB ID: 6K41[5]) and (b) partial agonist-bound α_{2A}AR (PDB ID: 6KUY[3]), viewed from the extracellular side. Potential steric clashes are indicated by black arrows. The receptor backbone is depicted as cartoon; (+)-cyclazosin is shown as sticks and as van der Waals spheres (an alternative orientation observed for its furan-2-yl-methanone substituent has been omitted for clarity, cf. Fig. 2a); $F^{7.39}$ is shown as sticks and as van der Waals spheres; the agonistic ligands dexmedetomidine and RES are shown as sticks.

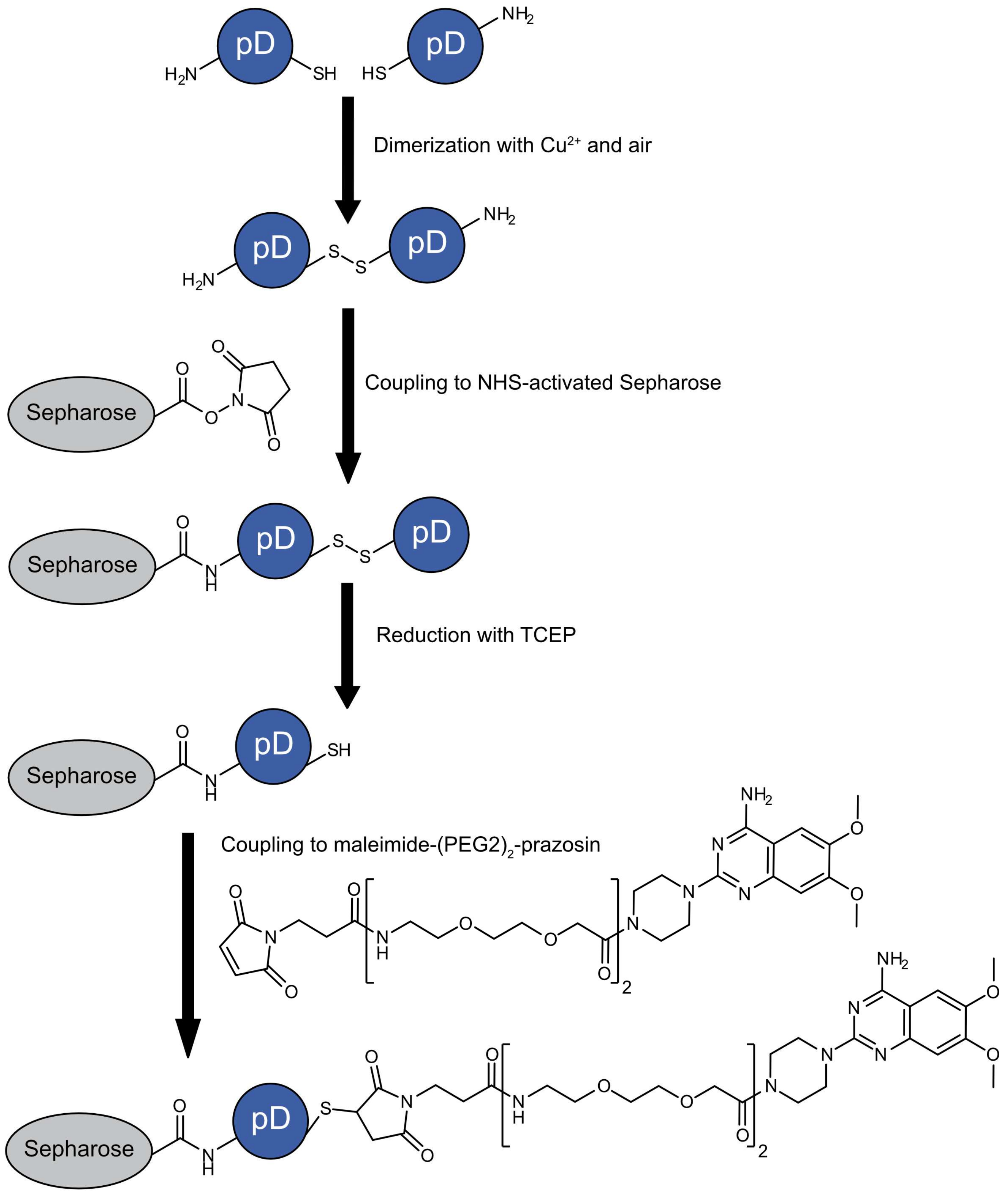

Supplementary Fig. 14. Schematic illustration of the prazosin ligand-affinity column and its preparation. pD, protein D (see Methods).

Supplementary Tables

Supplementary Table 1. Amino acid mutations harbored by the stabilized crystallization construct, referred to as $\alpha_{1B}AR_{XTAL}$, compared to wild-type (wt) $\alpha_{1B}AR$.

Residue number*	wt $\alpha_{1B}AR$	$\alpha_{1B}AR_{XTAL}$
95 (2.54)	S	C
116 (3.23)	I	T
124 (3.31)	V	M
150 (34.50, ICL2)	S	Y
168 (4.48)	S	C
183 (4.63/ECL2)	G	V
191 (ECL2)	D	Y
194 (ECL2)	E	V
295 (6.36)	T	M
333 (7.38)	V	L
334 (7.39)	F	L
349 (7.54)	P	L

*Ballesteros-Weinstein numbering[2] is indicated in parentheses.

Supplementary Table 2. Phenylephrine-induced G_q signaling in cells expressing wild-type α_{1B}AR or the constructs harboring the individual mutation indicated.

Construct	EC_{50} (log M)	E_{max} (% of α_{1B}AR)
α_{1B}AR	−6.52 ± 0.11 (6) [−6.75 to −6.29]	100.0 (6)
$S95^{2.54}C$	−5.78 ± 0.16 (3) [−6.12 to −5.44]	71.9 ± 5.7 (3) [60.1 to 83.6]
$I116^{3.23}T$	−6.84 ± 0.19 (3) [−7.24 to −6.44]	71.2 ± 6.4 (3) [57.8 to 84.6]
$V124^{3.31}M$	−6.76 ± 0.16 (3) [−7.09 to −6.42]	84.2 ± 6.3 (3) [71.0 to 97.4]
$S150^{34.50}Y$	n.a. (3)	13.4 ± 1.7 (3) [9.91 to 17.0]
$S168^{4.48}C$	−6.45 ± 0.18 (3) [−6.83 to −6.08]	96.8 ± 8.0 (3) [80.1 to 113.5]
$G183^{4.63/ECL2}V$	n.a. (3)	9.0 ± 18.5 (3) [−29.4 to 47.4]
$D191^{ECL2}Y$	−5.58 ± 0.11 (3) [−5.80 to −5.36]	89.6 ± 4.7 (3) [79.8 to 99.4]
$E194^{ECL2}V$	−6.61 ± 0.19 (3) [−7.00 to −6.22]	85.4 ± 7.4 (3) [70.0 to 100.7]
$T295^{6.36}M$	−5.67 ± 0.24 (4) [−6.15 to −5.18]	50.7 ± 5.9 (4) [38.6 to 62.8]
$V333^{7.38}L$	−5.57 ± 0.13 (4) [−5.83 to −5.30]	79.8 ± 5.2 (4) [69.2 to 90.5]
$F334^{7.39}L$	n.a. (4)	n.a. (4)
$P349^{7.54}L$	−5.82 ± 0.31 (4) [−6.46 to −5.18]	27.3 ± 4.1 (4) [18.9 to 35.7]

Data are shown as mean values ± SEM from 3–6 independent experiments performed in technical duplicates. The number of independent experiments is indicated in parentheses. The 95% confidence interval of the mean is given in square parentheses. n.a., non-applicable (cf. Supplementary Fig. 3b, c). Ballesteros-Weinstein numbering[2] is denoted in the superscript. Source data are provided as a Source Data file.

Supplementary Table 3. Data collection and refinement statistics.

Ligand	(+)-Cyclazosin
PDB code	7B6W
**Data collection**	
Space group	$P2_12_12_1$
Cell dimensions	
a, *b*, *c* (Å)	67.88
	76.42
	151.63
α, β, γ (°)	90
	90
	90
Resolution (Å)	28.71–2.87
	(3.07–2.87)
R_{merge}	0.77 (5.99)
R_{pim}	0.32 (2.62)
$I/\sigma(I)$	5.5 (1.7)
$CC_{1/2}$	0.62 (0.16)
Completeness (%)	89.0 (47.0)
Redundancy	6.9 (7.3)
**Refinement**	
Resolution (Å)	28.71–2.87
No. reflections	14777 (724)
Mol/ASU	1
R_{work}/R_{free}	0.288/0.317
No. atoms	
Protein	3320
Ligand	31
Detergent	-
H_2O	-
R.m.s. deviations	
Bond lengths (Å)	0.01
Bond angles (°)	1.67

Statistics were obtained from the STARANISO server[10] (see Methods). The geometrical quality of the model is reported in the Methods section. Mol/ASU: molecules per asymmetric unit. R.m.s.: root-mean-square. Highest resolution shell is shown in parentheses. Anisotropy-correction using the STARANISO server[10] resulted in a dataset extending to 2.87 Å with low completeness and poor data collection statistics in the highest resolution shell. However, better electron density maps and refinement statistics were obtained with the 2.87-Å anisotropy-corrected dataset compared to the 3.1-Å isotropically processed dataset. Refinement of the cyclazosin-$\alpha_{1B}AR_{XTAL}$ complex was performed with both the 2.87-Å and 3.1-Å datasets, and yielded very similar structures. The electron density of the 2.87-Å anisotropy-corrected dataset is of good quality (see main text and Supplementary Fig. 5). The 2.87-Å resolution cut-off was determined by the STARANISO server[10] — since $CC_{1/2}$ is not suitable as a metric for anisotropic data, only the mean $I/\sigma(I)$ was used, as explained in the Frequently Asked Questions section of the STARANISO server (https://staraniso.globalphasing.org/staraniso_FAQ.html). In addition, as explained in supplementary ref. 11, note that R_{merge} and R_{pim} may not be suitable indicators for the present dataset, which was obtained by merging several partial datasets from different crystals (see Methods).

Supplementary Table 4. Intermolecular interactions formed between (+)-cyclazosin and $\alpha_{1B}AR_{XTAL}$.

Ligand moiety	Interaction	$\alpha_{1B}AR_{XTAL}$ residue
OCH_3 at C6 of quinazoline ring	vdW	F311$^{6.52}$, L314$^{6.55}$
OCH_3 at C6 of quinazoline ring	vdW / weak polar	S207$^{5.42}$ [OH; 3.5]
OCH_3 at C7 of quinazoline ring	vdW	C129$^{3.36}$, W307$^{6.48}$, F310$^{6.51}$, F311$^{6.52}$
Quinazoline ring	vdW	A122$^{3.29}$, V126$^{3.33}$, F310$^{6.51}$, F311$^{6.52}$
Quinazoline ring	vdW	L334*$^{7.39}$, F334$_{MD}^{7.39}$
Quinazoline ring	aromatic	F310$^{6.51}$, F334$_{MD}^{7.39}$
NH_2 at C4 of quinazoline ring	H-bond	Y203$^{5.38}$ [OH; 3.0]
Protonated N1 of quinazoline ring	H-bond, salt bridge	D125$^{3.32}$ [OD2; 3.0]
Quinazoline ring (delocalized +-charge)	vdW / weak polar	D125$^{3.32}$ [OD2; 3.6], Y203$^{5.38}$ [OH; 3.5]
Piperazine ring	vdW	W121$^{3.28}$, A122$^{3.29}$, L334*$^{7.39}$, F334$_{MD}^{7.39}$
Fused cyclohexane ring	vdW	C118$^{3.25}$, W121$^{3.28}$, A122$^{3.29}$
Furan ring	vdW	L105$^{2.64}$, W121$^{3.28}$, K331$^{7.36}$
Furan ring	vdW	L334*$^{7.39}$, F334$_{MD}^{7.39}$, W335$^{7.40}$
Furan ring	aromatic	W121$^{3.28}$, F334$_{MD}^{7.39}$, W335$^{7.40}$

For polar interactions, the receptor atoms mediating the interaction and the distance in Å to the interacting ligand atoms are indicated in square parentheses (cut-off: 3.6 Å; No angle terms were considered for H-bonds). * indicates a mutation of $\alpha_{1B}AR_{XTAL}$ (F334 in wild-type $\alpha_{1B}AR$). MD in subscript indicates that the side chain of F334 was modeled by molecular dynamics simulations. Van der Waals interactions are abbreviated as vdW (cut-off: 4.5 Å). The term "aromatic interaction" is used as described in supplementary ref. 12. The analysis was based on Arpeggio, a web server for calculating and visualizing interatomic interactions in protein structures (http://biosig.unimelb.edu.au/arpeggioweb)[13] and on LigPlot+[14].

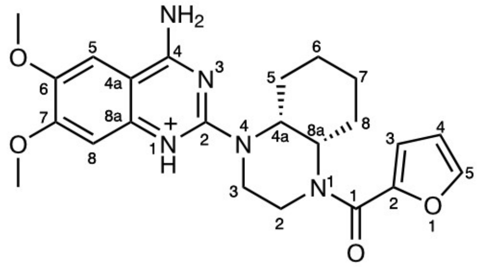

Supplementary Table 5. Intermolecular interactions formed between RS79948 and α_{2C}AR (PDB ID: 6KUW[4]).

Ligand moiety	Interaction	α_{2C}AR residue
OCH_3 at C3	vdW	V132$^{3.33}$
OCH_3 at C3	vdW / weak polar	S218$^{5.46}$ [OH; 3.2]
Benzene ring	vdW	V132$^{3.33}$, C135$^{3.36}$, L204$^{45.52}$, F398$^{6.51}$, F399$^{6.52}$, Y402$^{6.55}$
Benzene ring	aromatic	F398$^{6.51}$, F399$^{6.52}$, Y402$^{6.55}$
C5	vdW	C135$^{3.36}$, W395$^{6.48}$, F399$^{6.52}$
C6	vdW	W395$^{6.48}$, F398$^{6.51}$
Protonated N7	H-bond, salt bridge	D131$^{3.32}$ [OD1; 2.8], D131$^{3.32}$ [OD2; 3.2]
Protonated N7	cation-π	F398$^{6.51}$, F423$^{7.39}$
C13a	vdW	V132$^{3.33}$, F398$^{6.51}$
C13	vdW	L204$^{45.52}$, F398$^{6.51}$
C12a	vdW	F398$^{6.51}$, F423$^{7.39}$
C8	vdW	F398$^{6.51}$, F423$^{7.39}$
C8a	vdW	F423$^{7.39}$
C10	vdW	Y127$^{3.28}$, L128$^{3.29}$
C11	vdW	L128$^{3.29}$
C1	vdW	F423$^{7.39}$
C2	vdW	F419$^{7.35}$, F423$^{7.39}$

For polar interactions, the receptor atoms mediating the interaction and the distance in Å to the interacting ligand atoms are indicated in square parentheses (cut-off: 3.6 Å; No angle terms were considered for H-bonds). Van der Waals interactions are abbreviated as vdW (cut-off: 4.5 Å). The term "aromatic interaction" is used as described in supplementary ref. 12. The analysis was based on Arpeggio, a web server for calculating and visualizing interatomic interactions in protein structures (http://biosig.unimelb.edu.au/arpeggioweb)[13] and on LigPlot+[14].

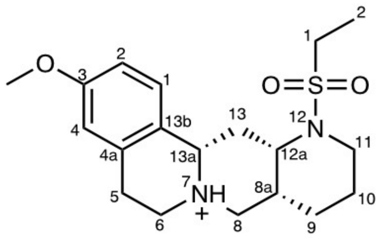

Supplementary Table 6. Affinities of QAPB, RS79948, prazosin, and cyclazosin for α_{1B}AR, α_{2C}AR, and chimeric α_{1B}AR-α_{2C} mutants.

Construct	Expr. (% of α_{1B}AR)	Sat. binding, pK_D	Comp. binding, pK_i		
		QAPB	RS79948	prazosin	cyclazosin
α_{1B}AR	100	8.11 ± 0.050 (8)<br>[8.00 to 8.23]	5.90 ± 0.037 (7)<br>[5.81 to 5.99]	9.47 ± 0.120 (3)<br>[8.93 to 10.01]	8.99 ± 0.067 (6)<br>[8.82 to 9.16]
α_{2C}AR	73 ± 9<br>[52 to 93]	6.86 ± 0.035 (6)<br>[6.77 to 6.95]	9.67 ± 0.078 (5)<br>[9.46 to 9.89]	7.39 ± 0.057 (3)<br>[7.14 to 7.63]	6.47 ± 0.071 (4)<br>[6.25 to 6.70]
α_{1B}AR-α_{2C}($Y^{3.28}$)	122 ± 5<br>[61 to 182]	7.88 ± 0.035 (4)<br>[7.77 to 8.00]	6.15 ± 0.084 (3)<br>[5.79 to 6.52]	n.d.	9.02 ± 0.098 (3)<br>[8.60 to 9.45]
α_{1B}AR-α_{2C}($L^{3.29}$)	144 ± 6<br>[124 to 164]	6.47 ± 0.034 (5)<br>[6.37 to 6.56]	8.04 ± 0.049 (5)<br>[7.91 to 8.18]	n.d.	8.49 ± 0.040 (5)<br>[8.38 to 8.60]
α_{1B}AR-α_{2C}($L^{45.52}$)	125 ± 3<br>[84 to 167]	7.95 ± 0.016 (4)<br>[7.90 to 8.01]	6.23 ± 0.020 (3)<br>[6.14 to 6.31]	n.d.	8.96 ± 0.036 (3)<br>[8.80 to 9.11]
α_{1B}AR-α_{2C}($Y^{6.55}$)	155 ± 8<br>[129 to 181]	7.38 ± 0.025 (7)<br>[7.32 to 7.44]	6.66 ± 0.036 (5)<br>[6.56 to 6.76]	n.d.	8.46 ± 0.062 (5)<br>[8.29 to 8.63]
α_{1B}AR-α_{2C}(YLLY)	193 ± 14<br>[159 to 227]	6.10 ± 0.100 (5)<br>[5.82 to 6.37]	8.78 ± 0.114 (4)<br>[8.41 to 9.14]	8.44 ± 0.10 (3)<br>[8.01 to 8.88]	8.43 ± 0.078 (4)<br>[8.18 to 8.67]
α_{1B}AR-α_{2C}(YLLY;ECL2)	121 ± 3<br>[114 to 127]	6.16 ± 0.150 (5)<br>[5.74 to 6.58]	8.02 ± 0.038 (4)<br>[7.91 to 8.15]	8.69 ± 0.10 (2)<br>[7.36 to 10.01]	8.57 ± 0.055 (3)<br>[8.33 to 8.81]
α_{1B}AR-α_{2C}(YLLY;$N^{2.64}$)	177 ± 5<br>[164 to 191]	6.06 ± 0.088 (6)<br>[5.84 to 6.29]	8.86 ± 0.093 (5)<br>[8.60 to 9.12]	8.18 ± 0.09 (3)<br>[7.77 to 8.58]	8.14 ± 0.052 (4)<br>[7.98 to 8.30]
α_{1B}AR-α_{2C}(YLLY;$N^{2.64}$;$G^{7.32}$)	215 ± 5<br>[200 to 231]	6.05 ± 0.066 (3)<br>[5.77 to 6.34]	8.57 ± 0.084 (2)	n.d.	8.13 ± 0.084 (2)
α_{1B}AR-α_{2C}(YLLY;$N^{2.64}$;$C^{5.43}$)	247 ± 17<br>[192 to 301]	6.04 ± 0.057 (3)<br>[5.80 to 6.29]	8.55 ± 0.059 (2)	n.d.	8.15 ± 0.140 (2)

Saturation ligand-binding of QAPB as well as competition ligand-binding with QAPB as the fluorescent tracer and the indicated unlabeled ligands as the competitor. Ligand-binding was measured on whole cells. Data are shown as mean values ± SEM from 3–8 independent experiments performed in technical triplicates, except for constructs α_{1B}AR-α_{2C}(YLLY;$N^{2.64}$;$G^{7.32}$) and α_{1B}AR-α_{2C}(YLLY;$N^{2.64}$;$C^{5.43}$), for which only two independent experiments were carried out. The exact number of independent experiments is indicated in parentheses. The 95% confidence interval of the mean is given in square parentheses for $n \geq 3$. n.d., not determined. Competition ligand-binding curves are shown in Supplementary Fig. 10. Differences in affinities were evaluated by a statistical test as shown in Supplementary Table 7. Expression levels were determined as described in the Methods section. Expr., expression; Sat., saturation; Comp., competition. Source data are provided as a Source Data file.

Supplementary Table 7. Significance of differences in affinities evaluated by one-way ANOVA and identification of significantly different pairs assessed with Dunnett's T3 statistical test for multiple comparisons assuming unequal variances.

Construct comparison		RS79948		cyclazosin		prazosin		QAPB	
		Sign.	P-value	Sign.	P-value	Sign.	P-value	Sign.	P-value
α_{1B}AR vs.	α_{2C}AR	****	<0.0001	****	<0.0001	**	0.0018	****	<0.0001
	α_{1B}AR-α_{2C}($Y^{3.28}$)	ns	0.2671	ns	>0.9999			*	0.0266
	α_{1B}AR-α_{2C}($L^{3.29}$)	****	<0.0001	**	0.0013			****	<0.0001
	α_{1B}AR-α_{2C}($L^{45.52}$)	***	0.0004	ns	0.9989			ns	0.0976
	α_{1B}AR-α_{2C}($Y^{6.55}$)	****	<0.0001	**	0.0015			****	<0.0001
	α_{1B}AR-α_{2C}(YLLY)	****	<0.0001	**	0.0054	**	0.0098	****	<0.0001
	α_{1B}AR-α_{2C}(YLLY;ECL2)	****	<0.0001			*	0.049	***	0.0004
	α_{1B}AR-α_{2C}(YLLY;$N^{2.64}$)	****	<0.0001	****	<0.0001	**	0.0038	****	<0.0001
	$\alpha_{1B}AR_{XTAL}$-ΔD12			**	0.0037				
	$\alpha_{1B}AR_{XTAL}$			ns	0.2082				
$\alpha_{1B}AR_{XTAL}$ vs.	$\alpha_{1B}AR_{XTAL}$-ΔD12			ns	0.5987				
α_{1B}AR-α_{2C}(YLLY) vs.	α_{2C}AR	***	0.0009						
	α_{1B}AR-α_{2C}($L^{3.29}$)	***	0.0008						
α_{1B}AR-α_{2C}(YLLY; $N^{2.64}$) vs.	α_{1B}AR-α_{2C}(YLLY)			ns	0.051	ns	0.3153		
	α_{2C}AR			****	<0.0001	*	0.0136		

Sign., significance; ns, not significant.

Supplementary References

1 Alexandrov, A. I., Mileni, M., Chien, E. Y., Hanson, M. A. & Stevens, R. C. Microscale fluorescent thermal stability assay for membrane proteins. *Structure* **16**, 351–359 (2008).

2 Ballesteros, J. A. & Weinstein, H. Integrated methods for the construction of three-dimensional models and computational probing of structure-function relations in G protein-coupled receptors. in *Methods in Neurosciences* Vol. **25**, 366–428 (Edited by Sealfon, S. C., Academic Press, 1995).

3 Qu, L. *et al.* Structural basis of the diversity of adrenergic receptors. *Cell Rep.* **29**, 2929–2935 (2019).

4 Chen, X. Y. *et al.* Molecular mechanism for ligand recognition and subtype selectivity of α_{2C} adrenergic receptor. *Cell Rep.* **29**, 2936–2943 (2019).

5 Yuan, D. *et al.* Activation of the α_{2B} adrenoceptor by the sedative sympatholytic dexmedetomidine. *Nat. Chem. Biol.* **16**, 507–512 (2020).

6 Moukhametzianov, R. *et al.* Two distinct conformations of helix 6 observed in antagonist-bound structures of a β_1-adrenergic receptor. *Proc. Natl. Acad. Sci. U. S. A.* **108**, 8228–8232 (2011).

7 Cherezov, V. *et al.* High-resolution crystal structure of an engineered human β_2-adrenergic G protein-coupled receptor. *Science* **318**, 1258–1265 (2007).

8 Wang, S. *et al.* Structure of the D2 dopamine receptor bound to the atypical antipsychotic drug risperidone. *Nature* **555**, 269–273 (2018).

9 Fan, L. *et al.* Haloperidol bound D_2 dopamine receptor structure inspired the discovery of subtype selective ligands. *Nat. Commun.* **11**, 1074 (2020).

10 Tickle, I. J. *et al.* STARANISO. http://staraniso.globalphasing.org (2018).

11 Karplus, P. A. & Diederichs, K. Assessing and maximizing data quality in macromolecular crystallography. *Curr. Opin. Struct. Biol.* **34**, 60–68 (2015).

12 Martinez, C. R. & Iverson, B. L. Rethinking the term "pi-stacking". *Chem. Sci.* **3**, 2191–2201 (2012).

13 Jubb, H. C. *et al.* Arpeggio: a web server for calculating and visualising interatomic interactions in protein structures. *J. Mol. Biol.* **429**, 365–371 (2017).

14 Laskowski, R. A. & Swindells, M. B. LigPlot+: multiple ligand-protein interaction diagrams for drug discovery. *J. Chem. Inf. Model.* **51**, 2778–2786 (2011).
