# Supplementary material for: Crystal structure of the α1B-adrenergic receptor reveals molecular determinants of selective ligand recognition
Source: Nat Commun. 2022 Jan 19;13:382. doi: 10.1038/s41467-021-27911-3 (PMC8770593; doi:10.1038/s41467-021-27911-3)
Supplement: Supplementary file 3 — Description of Additional Supplementary Files [file 41467_2021_27911_MOESM3_ESM.pdf]

Description of Additional Supplementary Files

File name: Supplementary Movie 1

Description: Superposition of the MD simulations of corynanthine-α2CAR (blue) and yohimbine-α2CAR (orange). The receptor backbone is shown as cartoon; the ligands and L1283.29 are depicted as sticks.
