# Supplementary material for: Crystal structure of the α1B-adrenergic receptor reveals molecular determinants of selective ligand recognition
Source: Nat Commun. 2022 Jan 19;13:382. doi: 10.1038/s41467-021-27911-3 (PMC8770593; doi:10.1038/s41467-021-27911-3)
Supplement: Supplementary file 5 — Reporting Summary [file 41467_2021_27911_MOESM5_ESM.pdf]

nature portfolio

Corresponding author(s): Andreas Plückthun, PhD
Daniel J. Scott, PhD

Last updated by author(s): Dec 2, 2021

Reporting Summary

Nature Portfolio wishes to improve the reproducibility of the work that we publish. This form provides structure for consistency and transparency in reporting. For further information on Nature Portfolio policies, see our Editorial Policies and the Editorial Policy Checklist.

Statistics

For all statistical analyses, confirm that the following items are present in the figure legend, table legend, main text, or Methods section.

n/a	Confirmed	
☐	☒	The exact sample size (*n*) for each experimental group/condition, given as a discrete number and unit of measurement
☐	☒	A statement on whether measurements were taken from distinct samples or whether the same sample was measured repeatedly
☐	☒	The statistical test(s) used AND whether they are one- or two-sided<br>*Only common tests should be described solely by name; describe more complex techniques in the Methods section.*
☒	☐	A description of all covariates tested
☐	☒	A description of any assumptions or corrections, such as tests of normality and adjustment for multiple comparisons
☐	☒	A full description of the statistical parameters including central tendency (e.g. means) or other basic estimates (e.g. regression coefficient) AND variation (e.g. standard deviation) or associated estimates of uncertainty (e.g. confidence intervals)
☐	☒	For null hypothesis testing, the test statistic (e.g. *F*, *t*, *r*) with confidence intervals, effect sizes, degrees of freedom and *P* value noted<br>*Give P values as exact values whenever suitable.*
☒	☐	For Bayesian analysis, information on the choice of priors and Markov chain Monte Carlo settings
☒	☐	For hierarchical and complex designs, identification of the appropriate level for tests and full reporting of outcomes
☒	☐	Estimates of effect sizes (e.g. Cohen's *d*, Pearson's *r*), indicating how they were calculated

Our web collection on statistics for biologists contains articles on many of the points above.

Software and code

Policy information about availability of computer code

Data collection	X-ray diffraction data were collected using the SSX data acquisition suite available at the PXI-X06SA beamline of the Swiss Light Source at the Paul Scherrer Institute.
Data analysis	XDS (version Mar 15, 2019 BUILT=20190806); AIMLESS (version 0.7.7); CCP4 package (version 7.1.016); STARANISO webserver (version 2.3.47); PHASER (version 2.8.3); COOT (version 0.96); REFMAC (version 5.8.0267); MolProbity (implemented in PHENIX version 1.19.2-4158-000); PyMOL (version 2.4.0a0); GraphPad Prism Suite (version 8.4.3); Rosetta fixbb; ICM-Pro (version 3.9-1b); Desmond (version 6.0); Schrödinger (version 19.4); VMD (version 1.9.3); LigPlot+ (version 2.2); The Arpeggio webserver was accessed at http://biosig.unimelb.edu.au/arpeggioweb on November 3, 2020.

For manuscripts utilizing custom algorithms or software that are central to the research but not yet described in published literature, software must be made available to editors and reviewers. We strongly encourage code deposition in a community repository (e.g. GitHub). See the Nature Portfolio guidelines for submitting code & software for further information.

Data

Policy information about availability of data

All manuscripts must include a data availability statement. This statement should provide the following information, where applicable:

- Accession codes, unique identifiers, or web links for publicly available datasets
- A description of any restrictions on data availability
- For clinical datasets or third party data, please ensure that the statement adheres to our policy

Coordinates and structure factors for the complex of α1BARXTAL and (+)-cyclazosin have been deposited in the worldwide PDB under the following accession code: 7B6W [http://doi.org/10.2210/pdb7B6W/pdb]. All data needed to evaluate the conclusions of the paper are present in the main manuscript and/or in the

Supplementary Information. Source data are provided with this paper. Additional data supporting the findings of this paper are available from the corresponding authors upon reasonable request. A reporting summary for this paper is available as a Supplementary Information file.
Additional publicly available PDB entries mentioned in this paper: 6KUW [http://doi.org/10.2210/pdb6KUW/pdb]; 6KUX [http://doi.org/10.2210/pdb6KUX/pdb]; 6KUY [http://doi.org/10.2210/pdb6KUY/pdb]; 6K41 [http://doi.org/10.2210/pdb6K41/pdb]; 2YCW [http://doi.org/10.2210/pdb2YCW/pdb]; 2RH1 [http://doi.org/10.2210/pdb2RH1/pdb]; 4LDO [http://doi.org/10.2210/pdb4LDO/pdb]; 6IBL [http://doi.org/10.2210/pdb6IBL/pdb]; 5LW2 [http://doi.org/10.2210/pdb5LW2/pdb].

Field-specific reporting

Please select the one below that is the best fit for your research. If you are not sure, read the appropriate sections before making your selection.

☒ Life sciences ☐ Behavioural & social sciences ☐ Ecological, evolutionary & environmental sciences

For a reference copy of the document with all sections, see nature.com/documents/nr-reporting-summary-flat.pdf

Life sciences study design

All studies must disclose on these points even when the disclosure is negative.

Sample size	No sample size-calculation was performed. The biochemical experiments were carried out in the number of replicates indicated in the paper, which enabled a sufficiently reliable quantification of the experimental findings based on the statistical indicators detailed in the paper. All biochemical experiments were repeated at least two times with biologically independent samples (different cell passages), and nearly all experiments were repeated at least three times with biologically independent samples (different cell passages), which is comparable to other published studies. For X-ray crystallography, diffraction data from multiple crystals were collected (27 partial datasets), which enabled a reliable structure determination of the receptor as detailed in the paper.
Data exclusions	Ligand-binding and signaling data biased by an experimental mistake (e.g., a pipetting mistake) were excluded from analysis. These data have been labeled accordingly in the source data file and are present in the measurements related to Figs. 4a and 5a-c, Supplementary Figures 3a-e and 10, and Supplementary Tables 2 and 6.
Replication	The biochemical experiments were carried out in the number of replicates indicated in the paper. Briefly, all biochemical experiments were repeated at least two times with biologically independent samples (different cell passages), and nearly all experiments were repeated at least three times with biologically independent samples (different cell passages). All attempts at replication were successful. Crystallization has been successfully reproduced at least two times with diffracting crystals.
Randomization	This study did not allocate experimental groups. Thus, randomization was not required for the reported experiments.
Blinding	This study did not allocate experimental groups. Blinding is not necessary for structure determination and for the signaling and ligand-binding assays reported in this study. All the experiments needed full information about the experimental system and were most often carried out by the same investigator.

Reporting for specific materials, systems and methods

We require information from authors about some types of materials, experimental systems and methods used in many studies. Here, indicate whether each material, system or method listed is relevant to your study. If you are not sure if a list item applies to your research, read the appropriate section before selecting a response.

Materials & experimental systems

n/a	Involved in the study	
☒	☐	Antibodies
☐	☒	Eukaryotic cell lines
☒	☐	Palaeontology and archaeology
☒	☐	Animals and other organisms
☒	☐	Human research participants
☒	☐	Clinical data
☒	☐	Dual use research of concern

Methods

n/a	Involved in the study	
☒	☐	ChIP-seq
☒	☐	Flow cytometry
☒	☐	MRI-based neuroimaging

Eukaryotic cell lines

Policy information about cell lines

Cell line source(s)	HEK293T/17 cells were used, obtained from ATCC.
Authentication	No authentication was used, as the experiments depend only on transient transfection of a recombinant protein.

Mycoplasma contamination	Cell lines were routinely tested and are free from mycoplasma contamination.
Commonly misidentified lines (See ICLAC register)	No commonly misidentified cell lines were used in this study.
